# Itraconazole nanosuspension for oral delivery: Formulation, characterization and in vitro comparison with marketed formulation

**Published:** 2010

**Authors:** M. Nakarani, A.K. Misra, J.K. Patel, S.S. Vaghani

**Affiliations:** 1 Unison pharmaceuticals. Ahmedabad; 2 Pharmacy Dept.TIFAC-CORE in NDDS. The M.S.University of Baroda; 3 Nootan pharmacy college, Visnagar; 4 Smt. R. B. Patel Mahila Pharmacy College, Atkot, Gujarat, India

**Keywords:** Nanosuspension, Itraconazole, Pearl milling technique

## Abstract

**Background and the purpose of the study:**

Itraconazole is a poorly water soluble drug which results in its insufficient bioavailability. The purpose of the present study was to formulate Itraconazole in a nanosuspension to increase the aqueous solubility and to improve its formulation related parameters, dissolution and hence oral bioavailability.

**Methods:**

Itraconazole nanosuspension was prepared by pearl milling technique using zirconium oxide beads as a milling media, Poloxamer 407 as a stabilizer and glycerol as a wetting agent. Effects of various process parameters like, stirring time and the ratio of the beads were optimized by keeping drug:surfactant:milling media (1:3.0:50) as a constant initially and then optimized process parameters were used to optimize formulation parameters by 32 factorial designs. The optimized nanosuspension was lyophilized using mannitol (1:1 ratio) as a cryoprotectant. Nanosuspension was characterized by particle size and size distribution, drug content, scanning electron microscopy, differential scanning colorimetry and X-ray diffraction techniques.

**Results:**

Optimized nanosuspension showed spherical shape with surface oriented surfactant molecules and a mean particle diameter of 294 nm. There was no significant change in crystalline nature after formulation and it was found to be chemically stable with high drug content.

**Conclusion:**

The in vitro dissolution profile of the optimized formulation compared to the pure drug and marketed formulation (Canditral Capsule) by using 0.1N Hydrochloric acid as release medium showed higher drug release.

## INTRODUCTION

Itraconazole is an orally active triazole antimycotic agent, which is active against a broad spectrum of fungal species including *Cryptococcus*, *Candida*, *Aspergillus*, *Blastomyces* and *Histoplasma capsulatum* var. *capsulatum* (1, 2). It is a weak basic drug which is soluble in lipids (nOctanol/Water partition, 5.66 at pH of 8.1) and a pKa of 3.7 (3). Itraconazole is ionized only at a low pH, such as gastric juice and as a result on oral administration, the gastric acidity is required for adequate dissolution. The bioavailability of itraconalzole is known to be increased after a meal in comparison to the fasting state. Since the bioavailability of poorly water-soluble drugs can be influenced by interactions with food or by the physicochemical conditions of the gastrointestinal (GI) tract, oral preparation of itraconazole is commonly prescribed to be administered according to a fixed dosing schedule, especially, to be taken immediately after meals. The oral bioavailability of itraconazole is maximal when it is taken with a full meal.

Poor water solubility of drug molecules, insufficient bioavailability, fluctuating plasma levels and high food dependency are the most important and common problems with this drug. Major efforts have been made for the development of customized drug carriers to overcome the disappointing in vivo fates of the drug (4, 5). Hence, there is a growing need for a unique strategy that can tackle the formulation related problems associated with the delivery of hydrophobic drugs in order to improve their clinical efficacy and optimize their therapy with respect to pharmacoeconomics.

The dissolution rate of poorly water soluble-drugs often becomes a rate-limiting step in their absorption from GI tract (6, 7). Various solubilization methods have been used to increase the drug solubility and dissolution properties, including the use of surfactant, water-soluble carriers, polymeric conjugates, and solid dispersion.

Preparation of drugs in form of nanosuspensions has shown to be a more cost-effective and technically simpler alternative, particularly for poorly soluble drugs, and yield a physically more stable product than liposome dispersions (8–10). With this technique, the drug, dispersed in water, is grounded by shear forces to particles with a mean diameter in the nanometer range (100–1000nm). The fineness of the dispersed particles causes them to dissolve more quickly owing to their higher dissolution pressure and leads to an increased saturation solubility. This may enhance the bioavailability of drugs compared with other microparticular systems. If in vivo dissolution velocity of the drug particles is low enough, the drug nanosuspensions will have the passive targeting advantages of colloidal drug carriers (11).

The aim of this study was, to employ the nanosuspension technique to produce itraconazole nanoparticles for oral administration, thereby avoiding the use of harmful additives and enabling to enhance the saturation solubility, dissolution and oral absorption of itraconazole. The optimized nanosuspension formulation was evaluated for in vitro dissolution profile in comparison to the pure drug and marketed formulation (Canditral Capsule).

## MATERIAL AND METHODS

### 

#### Materials

Itraconazole was a gift from Intas pharmaceutical limited (India). Zirconium oxide beads were gifted from Sun Pharmaceutical Industries Ltd. (India).Poloxamer 407 was purchased from BASF (Germany). Glycerol and Mannitol were purchased from S.d fine chemicals (India).

#### Preparation of Nanosuspension

Itraconazole powder (1%w/v) was dispersed in an aqueous solution containing glycerol (2.2%w/v) and different ratio of Poloxamer 407 in 20 ml vial. The resulting coarse pre-dispersion was comminuted using zirconium oxide beads (milling media) on a magnetic stirrer. Zirconium oxide beads were used in the preparation of nanosuspension due to their low cost and easy availability for lab scale production of nanosuspension in comparison to silver beads. Various parameters like the effect of stirring time and ratio of different size of zirconium oxide beads were optimized by keeping the drug: surfactant: milling media volume (1:3:50) as constant initially, then the optimized conditions of stirring time and ratio of different size of zirconium oxide beads were used throughout the study to optimize concentration of Poloxamer 407 and volume of milling media using 32 factorial designs to achieve minimum particle size (Table 1). The stirring was continued for 24 hrs at 750 rpm for the preparation of optimized nanosuspension formulation. The optimized formulation was lyophilized using mannitol as a cryoprotectant (1:1 ratio). Lyophilized nanosuspension was used for further study.

**Table 1:** 3^2^ factorial design lay out for preparation of Itraconazole nanosuspension.

Batch No.	X_1_	X_2_
ITZ1	2.5	40
ITZ2	2.5	50
ITZ3	2.5	60
ITZ4	3.0	40
ITZ5	3.0	50
ITZ6	3.0	60
ITZ7	3.5	40
ITZ8	3.5	50
ITZ9	3.5	60

X_1_ , Concentration of stabilizer (Poloxamer 407) (%w/v).

X_2_ , % v/v of Milling Media (Zirconium oxide beads).

#### Particle size and Size distribution

The mean particle diameter and size distribution of the prepared nanosuspension was measured by laser diffraction technique using Malvern particle size analyzer, SM 2000. Nanosuspension was added to the sample dispersion unit, and stirred at 2000 rpm with magnet in order to reduce the interparticulate aggregation, and laser obscuration range was maintained between 10–20%. The average particle size was measured after performing the experiment in triplicates.

#### Scanning Electron Microscopy (SEM)

The lyophilized powder for nanosuspension formulation was kept in the sampling unit as a thin film and then photographs were taken at 100X and 200X magnification using Jeol Scanning Electron Microscope (Jeol, JSM-840 SEM Japan).

#### Differential Scanning Calorimetry (DSC)

The DSC thermograms of bulk Itraconazole powder and lyophilized nanosuspension formulation were taken on a Mettler Toledo Star SW 7.01 DSC differential scanning colorimeter between 30–300°C at a heating rate of 10°C/min with Nitrogen supply at 50.0 ml/min.

#### X-ray Diffraction pattern (XRD)

The study was carried out at Punjab University, Chandigadh, India. The XRD thermograms of Bulk Itraconazole powder and lyophilized nanosuspension formulation were carried on Philips PW 1710 X-ray generator (Philips, Amedo, the Netherlands).

#### In vitro dissolution profile

In vitro dissolution study was performed using USP dissolution test apparatus-I (basket assembly). The dissolution was performed using 500 ml of 0.1N HCl and 900 ml phosphate buffer solution (PBS) of pH 6.8 as dissolution mediums maintained at 37±0.5°C and 100 rpm for pure drug, lyophilized itraconazole nanosuspension formulation and marketed formulation (Canditral capsule). Samples (5ml) were withdrawn at regular intervals of 5 min for 60 min and replaced with fresh dissolution medium. Samples were filtered through 0.2µ filter paper and assayed spectrophotometrically on SHIMADZU UV-VISIBLE spectrophotometer UV-1601 at 255.0 nm wavelength. Dissolution for each formulation was performed in triplicates and mean of absorbance was used to calculate cumulative percent of drug release (12).

#### Drug Content

Assay was carried out by taking 10 mg of lyophilized powder (weigh equivalent to 1.25 mg of drug), dissolved in 0.4 ml of tetrahydrofuran in 50 ml dry volumetric flask and then volume was made up using 0.1 N HCl. Then 4 ml of the solution was taken to 10 ml dry volumetric flask, and volume adjusted with 0.1 N HCl. The absorbance at 255.0 nm wavelength was taken using SHIMADZU UV-VISIBLE spectrophotometer UV-1601 and the drug content was calculated accordingly (13).

## RESULTS AND DISCUSSION

### 

#### Influence of various parameters on particle size and size distribution

As shown in Table 2, effect of stirring time on particle size was optimized by keeping 50:50 ratio of different diameter (0.4mm to 0.7mm and 1.2 mm to 1.7 mm) of zirconium oxide beads and keeping the drug: surfactant: milling media volume (1:3.0:50) constant. Lowest 317 nm mean particle size was achieved after 24 hrs stirring of 50:50 ratios of zirconium oxide beads. Further stirring up to 28 hrs may lead to increased particle size due to increased surface free energy.

**Table 2:** Effect of stirring time on particle size of itraconazole nanosuspension.

Batch. No.	Time (hrs)	Mean particle size [D(4, 3)]
IT1	Initial (5min.)	176.49µm
IT2	2	3.060µm
IT3	4	2.328µm
IT4	6	1.824µm
IT5	8	1.368µm
IT6	10	1.309µm
IT7	12	1.225µm
IT8	24	0.317µm
IT9	26	0.552µm
IT10	28	0.678µm

As shown in Table 3, the effect of ratio of different size of zirconium oxide beads from 0.4 nm to 0.7 nm and 1.2 nm to 1.7 nm on particle size was optimized by keeping the drug: surfactant: milling media volume (1:3.0:50) constant and stirring for 24 hrs. Lowest particle size of 315 nm was observed at 50: 50 resulting ratio of different size of zirconium oxide beads. When the ratios of different size of zirconium oxide beads were different than 50:50, resulting nanosuspensions had higher particle size (Table 3). One possible explanation is that at this ratio beads were closely packed and lead to reduced void space between various size beads. At different ratios other than this, the void spaces were found to be higher and attrition between drug particles and beads were at maximum.

**Table 3:** Effect of Ratio of beads on particle size of itraconazole nanosuspension

Batch. No.	Ratio of beads (Zirconium Oxide)		

	Small Size (0.4mm to 0.7mm)	Big Size (1.2mm to 1.7mm)	Mean particle size [D^4^, ^3^)]
TSB1	0	100	1.142µm
TSB2	25	75	0.674µm
TSB3	50	50	0.315µm
TSB4	75	25	0.865µm
TSB5	100	0	1.315µm

As shown in Table 4, the optimized formulation showed mean particle size of 283 nm with Polydispersity index of 0.307 (before lyophilization), with 3.0% w/v of poloxamer 407 which was used as a stabilizer and 50% v/v of milling media. After lyophilization a mean particle diameter was found to be 294 nm with Polydispersity index 0.318, so in lyophilization process there was no significant change in particle size and size distribution.

**Table 4:** Optimization of formulation parameters for the preparation of itraconazole nanosuspension.

Batch No.	Conc. of drug (% w/v)	Conc. stabilizer (Poloxamer 407) (% w/v)	% v/v o f Milling Media (Zirconium oxide beads)	Particle size before Ly o p h i l i z a t i o n [d(4, 3)]	Polydispersity index	Particle size after Lyophilization [d(4, 3)]	Polydis persity index
ITZ1	1	2.5	40	0.747µm	0.512	0.754µm	0.523
ITZ2	1	2.5	50	0.516µm	0.419	0.523µm	0.428
ITZ3	1	2.5	60	0.696µm	0.489	0.707µm	0.508
ITZ4	1	3.0	40	0.379µm	0.376	0.392µm	0.389
ITZ5	1	3.0	50	0.283µm	0.307	0.294µm	0.318
ITZ6	1	3.0	60	0.324µm	0.453	0.358µm	0.461
ITZ7	1	3.5	40	0.501µm	0.392	0.515µm	0.402
ITZ8	1	3.5	50	0.438µm	0.543	0.448µm	0.554
ITZ9	1	3.5	60	0.492µm	0.465	0.505µm	0.481

No significant changes in particle size and polydispersity index demonstrate formation of stable non-flocculated nanosuspension of itraconazole which was developed in this investigation (Table 4). Lowest mean particle size was achieved after 24 hrs stirring with milling media ratio of 0.4–0.7 mm and beads of diameter 1.2–1.7 mm at 50:50. Increase in the media volume led to slight increase in the mean particle diameter but did not lead to significant change by increasing the concentration of stabilizer. Further stirring resulted in increased mean particle diameter of Itraconazole nanosuspension which may be due to increased surface free energy.

#### Scanning electron microscopy (SEM)

**Figure 1:**
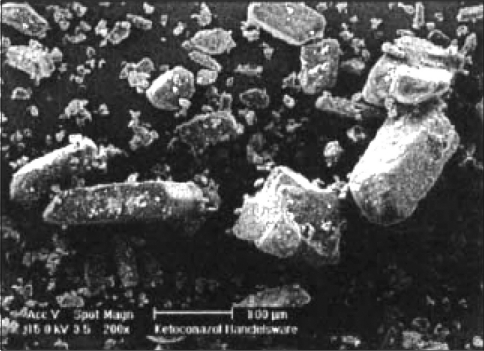
Photomicrograph of scanning electron micrographs of pure itraconazole powder.

**Figure 2:**
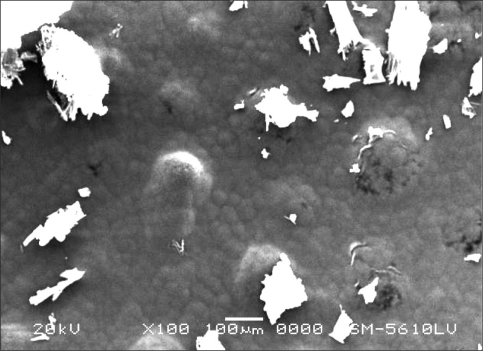
Photomicrograph of scanning electron micrographs of itraconazole nanosuspension formulation.

SEM micrographs clearly showed great differences between pure itraconazole (Figure. 1) and optimized nanosuspension formulation (Figure. 2). The particles of itraconazole were found to be large and especially irregular (Figure. 1). However after formulation, particles disappeared and drug became small and uniform. The nanocrystals seem to be more rounded, perhaps because the particles were coated with a surfactant layer. In the suspension suolution, the surfactant which was used to stabilize the particles could be adsorbed to surface of the crystals by hydrophobic interaction. Therefore, after lyophilization of surfactants an amorphous layer formed on the surface of inner crystals. (14)

#### Differential Scanning Calorimetry (DSC)

**Figure 3:**
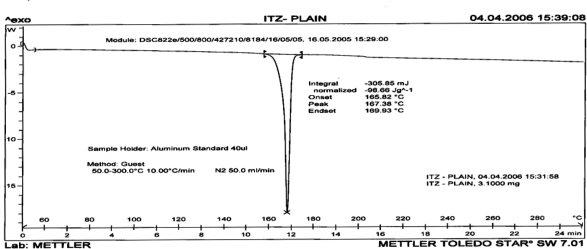
DSC thermograms of bulk Itraconazole powder

**Figure 4:**
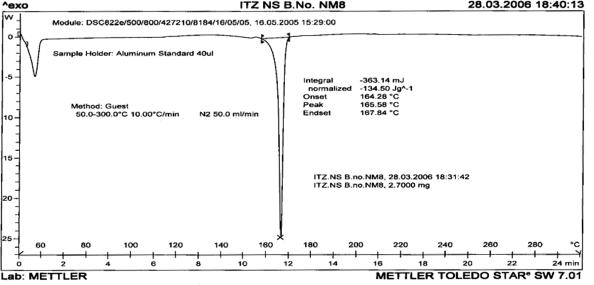
DSC thermograms of Itraconazole nanosuspension formulation.

DSC was performed to investigate the effect of surfactant on the inner structure of itraconazole nanosuspension. Figures 3 and 4 show DSC thermograph of pure itraconazole powder and optimized nanosuspension formulation respectively. Pure itraconazole powder showed melting exotherm at 168.38°C which corresponds to its melting point and its exotherm in formulation was observed at 165.58°C. From thermograms, it was concluded that the drug and the surfactant do not interact with each other (15).

#### X-Ray Diffraction pattern (XRD)

**Figure 5:**
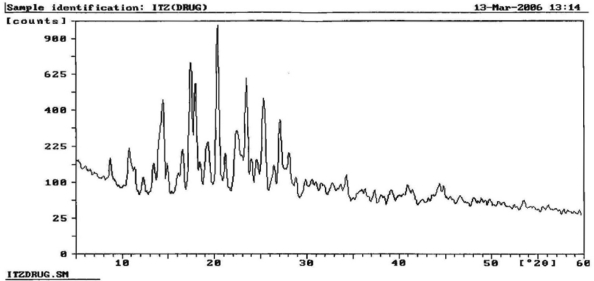
XRD thermograms of bulk Itraconazole powder.

**Figure 6:**
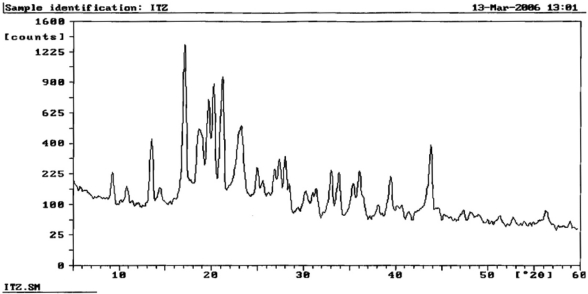
XRD thermograms of itraconazole nanosuspension formulation.

X-Ray diffraction was used to analyze potential changes in the inner structure of itraconazole nanocrystal during the formulation. The extent of such changes depends on the chemical nature and physical hardness of the active ingredient (16). Figures 5 and 6 show XRD thermograph of pure itraconazole powder, poloxamer 407, mannitol and itraconazole nanosuspension formulation respectively. The obtained patterns reveal that the drug crystallanity of nanosuspension formulation was not affected significantly (15).

#### In vitro dissolution study

**Figure 7:**
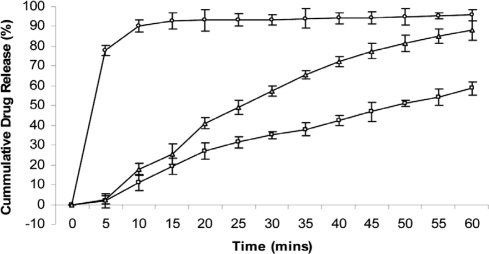
Dissolution profile for nanosuspension formulation (circle), pure drug (square), and marketed formulation (triangle) [mean ± SD (n=3)] in 0.1 N HCl.

**Figure 8:**
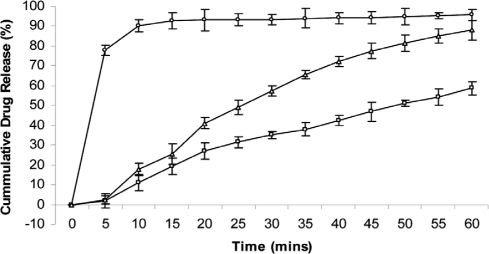
Dissolution profile for nanosuspension formulation (circle), pure drug (square), and marketed formulation (triangle) [mean ± SD (n=3)] in PBS pH 6.8.

Dissolution studies were performed for pure drug, marketed formulation (Canditral capsule) and optimized nanosuspension formulation. The amount of drug released from the optimized nanosuspension formulation was 90% within 10 min compared to amount of 10% and 17% for pure drug and marketed formulation (Canditral capsule) respectively (Figure 7) in 0.1N HCl (pH 1.2) while in PBS (pH 6.8) it was 92% as compared to 13% and 20% for pure drug and marketed formulation (Canditral capsule) respectively (Figure 8). The increase in accessible surface area to the dissolution medium and hydrophilic surfactant coating on the particle surfaces may be the reason for six fold increase in dissolution rate.

The drug content in the formulation was found to be 99.25% w/w of the amount of drug which was added theoretically. In the formulation no step was involved which could cause the drug loss hence a high amount of drug was obtained.

## CONCLUSION

From the results of this study it may be concluded that nanocrystalline suspensions of poorly soluble drugs such as itraconazole are easy to prepare and to lyophilize for extended storage and represent a promising new drug formulation for oral drug delivery for treatment of fungal infection. Dissolution study in 0.1N HCl shows that nanosuspension formulation gives higher drug release compared to the pure drug and marketed formulation. Consequently nanosuspensions represent a promising alternative to current delivery systems aiming to improve the biopharmaceutic performance of drugs with low water solubility.

## References

[1] Saag MS, Dismukes WE (1988). Azole antifungal agents: emphasis on new triazoles. Antimicrob Agents Chemother.

[2] Odds FC, Oris M, Dorsselaer PV, Gerven FV (2000). Activities of an intravenous formulation of itraconazole in experimental disseminated Aspergillus, Candida and Cryptococcus infections.. Antimicrob Agents Chemother.

[3] Fromtling RA (1987). Recent Trends in the Discovery, Development and Evaluation of Antifungal Agents.

[4] Barratt GM (2000). Therapeutic applications of colloidal drug carriers. Pharm Sci Tech Today.

[5] Amidon GL, Lennernas H, Shah VP, Crison JR (1995). A theoretical basis for a biopharmaceutic drug classification: the correlation of in vitro drug product dissolution and in vivo bioavailablility. Pharm Res.

[6] Maeda T, Takenaka H, Yamahira Y, Noguchi T (1979). Use of rabbits for GI drug absorption studies: relationship between dissolution rate and bioavailability of griseofulvin tablets. J Pharm Sci.

[7] Chiba Y, Kohri N, Iseki K, Miyazaki K (1991). Improvement of dissolution and bioavailability for mebendazole, an agent for human echinococcosis, by preparing solid dispersion with polyethylene glycol. Chem Pharm Bull.

[8] Liversidge ME, Sarpotdar P, Bruno J, Hajj S, Wel L (1996). Formulation and antitumor evaluation of nano crystalline suspensions of poorly soluble anticancer drug. Pharm Res.

[9] Muller RH, Peters K (1997). Nanosuspensions for the formulation of poorly soluble drugs I. Preparation by a size reduction technique. Int J Pharm.

[10] Westesen K, Siekmann B (1995). Preparation and physicochemical characterization of aqueous dispersions of coenzyme Q10 nanoparticles. Pharm Res.

[11] Muller RH (1991). In vivo distribution of carriers. Colloidal Carriers for Controlled Drug Delivery and Targeting.

[12] Mishra B, Arya N, Tiwari S (2010). Investigation of formulation variables affecting the properties of lamotrigine nanosuspension using factorial design. DARU.

[13] Guo J, Ping Q, Chen Y (2001). Pharmacokinetic behavior of Cyclosporine A in rabbits by oral administration of lecithin vesicle and sandimmun neoral. Int J Pharm.

[14] Muller RH, Jacobs C (2002). Buparvaquone mucoadhesive nanosuspension: preparation, optimization and longterm stability. Int J Pharm.

[15] Teeranachaideekul V, Junyaprasert VB, Souto EB, Muller RH (2008). Development of ascorbyl palmitate nanocrystals applying the nanosuspension technology. Int J Pharm.

[16] Muller RH, Jacobs C, Kayser O (2001). Nanosuspensions as particulate drug formulations in therapy. rationale for development and what we can expect for the future. Adv Drug Deliv Rev.

